# Integrating Business Intelligence and CRM Systems With a Machine Learning Approach for Predictive Customer Retention in E‐Commerce

**DOI:** 10.1155/tswj/1946904

**Published:** 2026-05-27

**Authors:** Mohammad Zeinali, Leila Ramezani Asli, Mohammad Amin Khalili

**Affiliations:** ^1^ Faculty of Engineering, University of Isfahan, Isfahan, Iran, ui.ac.ir; ^2^ Department of Industrial Engineering, Tafresh University, Tafresh, Iran, tafreshu.ac.ir; ^3^ Department of Earth, Environmental and Resource Sciences, Monte Sant′Angelo Campus, Federico II University of Naples, Naples, Italy, unina.it; ^4^ School of Geography, Geology and the Environment, University of Leicester, Leicester, UK, le.ac.uk

**Keywords:** business intelligence (BI), churn prediction, customer relationship management (CRM), customer segmentation, e-commerce data analytics

## Abstract

In the rapidly evolving e‐commerce landscape, retaining existing customers has become more cost‐effective and strategically important than acquiring new ones. This study proposes a data‐driven framework that integrates business intelligence (BI) tools, machine learning, and customer relationship management (CRM) decision support to improve predictive customer retention. The framework was developed using the publicly available Brazilian E‐Commerce Public Dataset (Olist), which contains more than 100,000 orders and includes transactional, payment, delivery, product, and customer‐review information. After SQL‐based integration and feature engineering, customer segmentation was performed using *K*‐means clustering on recency, frequency, monetary (RFM) variables, identifying three behavioral groups: loyal, at‐risk, and occasional customers. For churn prediction, Random Forest and XGBoost classifiers were trained on customer‐level behavioral, satisfaction, and service‐related features. XGBoost achieved the best overall performance, with accuracy = 0.81, precision = 0.79, recall = 0.83, *F*1 − score = 0.81, and AUC = 0.85, outperforming Random Forest (accuracy = 0.76, precision = 0.74, recall = 0.71, *F*1 − score = 0.72, and AUC = 0.76). The resulting segmentation and churn scores were then exposed through Power BI dashboards and mapped into a proof‐of‐concept CRM decision framework for retention planning. Unlike studies that treat BI, machine learning, or CRM in isolation, this research presents an end‐to‐end analytical pipeline that links data preparation, predictive modeling, dashboard‐based decision support, and scenario‐level CRM action design. The framework provides a reproducible basis for e‐commerce retention analytics and a practical foundation for future live deployment and A/B‐tested CRM validation.

## 1. Introduction

In the competitive digital economy, e‐commerce is one of the most dynamic and fast‐expanding sectors. Online markets, mobile commerce, and digital payment platforms have changed consumer behavior, corporate operations, and marketing techniques. As worldwide internet availability and digital touchpoints rise, e‐commerce platforms are proliferating. This increase raises customer expectations and market saturation, making customer acquisition expensive and unsustainable. Recent industry studies say getting a new customer costs five times more than retaining one [1]. Customer retention is a key component of e‐commerce digital marketing strategy, affecting profitability, CLV, and brand loyalty (S. [2]).

Despite this, many e‐commerce businesses focus too much on acquisition‐based efforts and ignore the rich insights that may be gained from customer data to build loyalty and engagement. A gap in how firms use business intelligence (BI) and customer relationship management (CRM) systems synergistically to improve retention motivated this research [3]. These tools are valuable separately, but their combined use—especially in data‐driven digital marketing—is underexplored in theory and practice [4].

E‐commerce customer retention requires long‐term relationships, unique experiences, and consistent participation. Customers who stay are more likely to suggest the brand, give great reviews, and spend more over time, which boosts sales. Digital marketing retention tactics include email campaigns, loyalty programs, retargeting ads, and personalized product suggestions [5].

Due to fragmented or unused customer data, digital marketing struggles with personalization and segmentation. Many platforms do not track long‐term behavioral trends or have the analytics to create retention strategies [6]. Generic campaigns cause customer fatigue, disengagement, and churn. Digital marketing efforts lack feedback loops, making retention strategy optimization difficult. These shortcomings require more intelligent, data‐driven systems to assess customer behavior and adjust marketing tactics [7].

Integration of BI and CRM systems helps fill the gaps by turning raw customer data into actionable insights (Nnaemeka Stanley [8]). Companies may evaluate consumer purchase habits, discover churn indicators, and track marketing campaign performance in real‐time using BI tools like Power BI, Tableau, and Google Data Studio. However, CRM systems are the operational layer that lets organizations categorize clients, personalize communication, and track engagement history.

These technologies are often employed alone in small‐ to medium‐sized e‐commerce enterprises, limiting their impact. CRM is often limited to a contact database with basic automation, and BI dashboards are utilized only for sales reports, not predictive and prescriptive marketing. This segmented approach hampers current e‐commerce [9]. Combining BI′s descriptive and diagnostic capabilities with CRM′s interactive and executional strengths allows firms to create data‐driven, customer‐centric retention strategies [10].

This study focuses on improving customer retention techniques in e‐commerce digital marketing by bridging the operational and analytical gap between BI tools and CRM systems. Today′s data‐rich e‐commerce environment gives firms access to vast client data, yet many fail to use it for marketing [11]. This study uses the Brazilian E‐Commerce Public Dataset (Olist) to show how BI tools can be used for real‐time decision‐making and retrospective analysis [12]. The study shows how e‐commerce organizations can strengthen customer retention using advanced machine learning models for segmentation and churn prediction [13].

This research has three goals. First, it assesses how well BI tools can derive valuable insights from consumer contact data, including buying behavior, order frequency, and satisfaction. Second, it wants to use machine learning methods like clustering for segmentation and classification models for churn prediction to turn transactional data into predictive patterns. Third, it proposes a CRM framework integrating BI‐derived data for targeted marketing, proactive customer involvement, and optimization of loyalty campaigns. The ultimate goal is to show how data science can bridge research and action, enabling marketers to shift from intuition‐based campaign decisions toward evidence‐driven, anticipatory retention management. The following research questions assist the study in achieving these goals. How can BI technologies discover consumer behavior patterns that indicate retention or churn? What machine learning models best use behavioral and transactional data for e‐commerce client segmentation? How can CRM platforms use these analytical insights to improve digital marketing campaign targeting, timing, and relevance to support customer retention? The study′s methodology is based on these issues and contributes to academic inquiry and data‐driven e‐commerce marketing practice [14].

For terminology consistency throughout the manuscript, customer churn is used to denote the predictive event of customer disengagement or defection, whereas customer retention refers to the managerial objective and the associated CRM and marketing strategies designed to prevent churn; the term customer attrition is treated as synonymous with churn and is avoided in the remainder of the paper.

The remainder of this paper is structured as follows: Section 2 reviews the relevant literature, Section 3 presents the theoretical framework, Section 4 describes the research methodology, Section 5 reports the results, Section 6 discusses the findings, and Section 7 presents the conclusion.

## 2. Literature Review

E‐commerce companies, where competition is fierce and switching costs are cheap, are prioritizing customer retention. Online marketplaces give customers instant access to many options, so businesses must provide consistent, engaging, and personalized experiences to build loyalty [15]. This endeavor centers on digital involvement. Engagement measures like click‐through rates, time spent on site, repeat visits, and feedback ratings reveal customer behavior and satisfaction. According to studies, retained customers have a better revenue share, lifetime value, and brand recommendation rate. Due to the mismatch between data analytics and customer‐facing initiatives, many firms fail to operationalize digital engagement in ways that promote retention [16].

BI can extract useful insights from complicated and massive e‐commerce transaction data. Companies use data warehousing, dashboards, visual analytics, and predictive modeling to make informed decisions based on historical and real‐time data. E‐commerce companies employ BI technologies to examine consumer purchase history, sales trends, inventory movement, and marketing campaign performance. These data can help identify at‐risk customers, assess satisfaction, and detect churn risk and retention‐related behavioral tendencies. While BI tools are becoming more popular, they are generally used for operational reporting rather than strategic decision‐making. BI′s ability to guide customer retention is underutilized when not integrated into customer interaction tools like CRM platforms [17].

CRM systems have grown from simple contact management tools to sophisticated platforms for managing and automating client interactions across digital media [18]. Modern CRM systems may track client histories, segment user groups, automate communication procedures, and integrate with social media and e‐commerce platforms. CRM systems power targeted advertising, engagement analytics, and consumer personalization in digital marketing. CRM systems support real‐time marketing as consumers want more relevance and immediacy from brands [19]. A significant issue is that many CRM implementations lack the analytical depth to prioritize or personalize activities. CRM systems might become passive repositories rather than dynamic customer retention tools without robust data inputs or analytical stacking. Thus, CRM solutions must be integrated with analytical engines like BI platforms to improve strategic value (Uloma Stella [20]).

The combination of BI and CRM platforms advances customer‐centric digital marketing. BI‐driven CRM models integrate data analytics with customer engagement workflows to personalize marketing campaigns with real‐time information. This synergy lets marketers transition from static segmentation and campaign planning to context‐aware interaction. BI dashboards integrated with CRM may automatically identify high‐risk customers and initiate retention efforts based on behavior or transaction patterns [21]. Recent theoretical models suggest that BI tools update CRM systems with new insights, enabling data‐driven decision‐making throughout the customer experience [22]. These models are primarily theoretical or tested exclusively in large companies with mature data infrastructures. These frameworks must be operationalized and validated in practical e‐commerce environments, especially for small and medium‐sized firms with limited resources [23].

Beyond the general BI–CRM literature, prior research on churn analytics offers several important foundations for the present study. [24] showed that customer retention is one of the most heavily studied CRM domains in data mining and highlighted classification, clustering, and association methods as central analytical tools. In predictive churn research, [25] demonstrated that bagging and boosting classification trees can substantially improve churn prediction performance, while [26] emphasized that churn datasets are often imbalanced and therefore require careful treatment through suitable evaluation metrics and learning strategies. [27] further extended churn modeling by showing that relational and social‐network information can improve prediction in settings where customer interactions influence defection behavior. From the BI perspective, [28] argued that BI and analytics create organizational value when predictive models are connected to decision processes rather than treated as isolated technical exercises. Compared with these streams, the present study contributes by combining e‐commerce churn prediction, customer segmentation, BI dashboarding, and CRM‐oriented retention logic within one end‐to‐end framework based on a real multitable transactional dataset.

Recent studies on customer churn prediction have increasingly applied machine learning to improve retention decisions, but most of this literature remains focused on predictive accuracy rather than deployment within a broader BI–CRM system. Many churn studies are conducted in telecommunications, subscription services, or general retail settings and typically rely on account history, tenure, usage intensity, or demographic variables, with the main contribution framed around classifier performance or explainability [29, 30]. In e‐commerce settings, prior work has shown that transactional and behavioral variables such as recency, frequency, monetary (RFM) value, and purchase history can support effective churn prevention, yet these studies often stop at model development and do not clearly demonstrate how model outputs are translated into decision‐support dashboards or CRM workflows [13, 31, 32]. At the same time, BI‐ and CRM‐oriented studies emphasize dashboarding, reporting, customer knowledge, and marketing automation, but often without an explicit churn modeling layer based on customer‐level machine learning features [18, 19]. This creates a practical research gap between prediction‐oriented churn analytics and action‐oriented CRM execution.

Accordingly, the existing literature may be grouped into three main streams: (1) machine learning–based churn prediction studies, which primarily optimize classification performance; (2) BI studies, which emphasize dashboarding, descriptive analytics, and managerial reporting; and (3) CRM studies, which focus on customer interaction, personalization, and workflow automation. What remains limited is an empirical end‐to‐end framework that connects these three layers in a single e‐commerce setting. The present study addresses this gap by using a real multitable e‐commerce dataset (Olist), engineering both classical behavioral variables and service‐experience features, applying machine learning for segmentation and churn prediction, and then translating those analytical outputs into BI‐supported CRM decision logic. Therefore, the contribution of this research is not merely the use of churn prediction models but the integration of predictive analytics, BI decision support, and CRM‐oriented retention design within one operationally interpretable framework suitable for e‐commerce organizations with limited technical resources.

## 3. Theoretical Framework

### 3.1. Retention Strategies Through BI and CRM Synergy

Keeping customers has been known for a long time to be an important part of making money in e‐commerce. Customers who stay with a business usually bring in more money over time, spend less on marketing, and promote the business through reviews and word of mouth. However, retention strategies work best when they are based on facts and are carried out precisely. This is why BI and CRM need to work together. BI provides the data needed to understand customer behavior, while CRM enables organizations to act on those insights by tailoring communications, streamlining processes, and maintaining a complete history of customer interactions.

When BI and CRM are combined, they create a feedback loop that turns real‐time insights into marketing choices that can be implemented [33]. BI tools help find trends, like fewer purchases, bad reviews, or changes in what categories people like, which could mean that a customer is leaving. When you feed these insights into CRM systems, they can take action by sending targeted retention campaigns, loyalty awards, or personalized re‐engagement emails specific to each customer profile. This integration ensures that customer engagement is not based on broad categories but on detailed, dynamically updated behavioral profiles. Therefore, BI–CRM integration‐based retention strategies are more flexible, up‐to‐date, and focused on the customer than traditional approaches based on intuition alone.

### 3.2. Conceptual Model of BI‐Driven CRM

This study suggests a way to arrange the combination of BI and CRM into a helpful model for customer retention [34]. It does this by connecting data analytics with marketing actions that CRM drives. The structure comprises three main parts: collecting and cleaning data, using analytics and insights to make decisions, and implementing those decisions using CRM. In the first layer, transactional, behavioral, and feedback data from e‐commerce sites are gathered and prepared for further processing. The second layer uses machine learning algorithms and visual analytics to divide customers into groups, estimate how likely those groups are to leave, and find the most valuable clients. For the last part, the third layer links these insights to CRM features like managing reward programs, sending automated emails, dividing customers into groups, and setting customer service priorities [35].

From a system‐architecture perspective, the proposed BI–CRM framework can be understood as a layered data flow. First, raw data from e‐commerce sources such as orders, payments, customer profiles, reviews, and product records are extracted and consolidated into a centralized analytical repository (i.e., a data warehouse or customer‐level analytical mart) through SQL‐based integration and preprocessing. Second, this integrated dataset is passed to the machine learning pipeline implemented in Python, where customer segmentation and churn prediction models generate outputs such as segment labels, churn probabilities, and retention priority scores. Third, these scored outputs are loaded into the BI layer (Power BI), which serves not only as a visualization environment but also as a decision‐support layer in which marketers can monitor risk, prioritize customer groups, and define intervention rules. Finally, the BI outputs can be transferred to a CRM action layer through scheduled exports, APIs, or middleware connectors, where campaign logic is operationalized into retention actions such as loyalty offers, re‐engagement emails, service recovery tasks, and follow‐up tracking. In this architecture, middleware acts as the integration bridge that maps model outputs to CRM fields, triggers workflow rules, and returns campaign‐response data to the BI environment, thereby closing the feedback loop.

**Figure 1:**
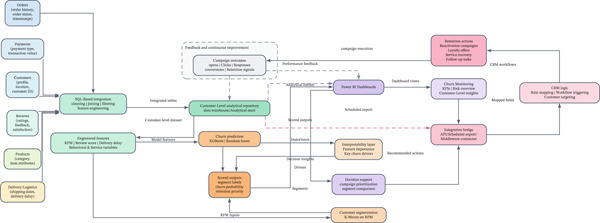
Conceptual BI–CRM integration architecture for customer retention, showing the flow from e‐commerce data sources to the analytical repository, machine learning pipeline, BI decision‐support layer, and CRM action layer.

Figure 1 shows that the model starts by importing data from several sources, such as payments, sales, customer information, and product reviews. BI tools like Power BI and Python‐based analytics environments are used to process these datasets and extract key metrics such as RFM values, customer satisfaction scores, and churn indicators. The results are then added to the CRM tool, which sets up and automates marketing actions based on how customers act in real‐time. For instance, customers who are likely to leave can be put into personalized retention processes immediately, and loyal customers can be targeted for referral campaigns or special deals. This model stresses the constant flow of data from analysis to action. This ensures that customer retention efforts remain adaptive and evidence‐driven.

### 3.3. Hypothesis Development

This study mainly uses an analytical and implementation‐based approach, but it is based on a set of hypotheses that help evaluate the suggested framework. The main idea behind the study is that adding BI analytics to CRM systems makes customer retention strategies much more successful in online shopping. This proposition holds that the predictive and descriptive power of BI tools directly improves the personalization, speed, and context‐relevance of CRM actions [36].

A second hypothesis is that machine learning–based segmentation and churn prediction outperform standard rule‐based methods in identifying high‐value segments and at‐risk customers. This is evaluated by comparing data‐driven model performance against basic threshold‐based benchmarks using accuracy, precision, recall, and *F*1‐score. A third proposition, framed as a direction for future research rather than a testable claim within this study, suggests that customer engagement metrics such as repeat purchase rates, interpurchase intervals, and positive review frequencies would improve following live deployment of BI‐informed CRM strategies. These hypotheses provide a framework for testing them in the real world and help improve the theory of digital marketing by showing how integrating analytics can help.

## 4. Research Methodology

### 4.1. Research Design and Workflow

This study uses an applied approach with several steps that connect descriptive analytics, predictive modeling, and strategic application in the context of keeping e‐commerce customers. We want to discover retention patterns in a large online retail dataset and how BI tools can effectively add predictive insights to CRM systems. For this reason, a relational e‐commerce dataset with sufficient volume, temporal coverage, and customer‐level behavioral detail was required so that segmentation, churn modeling, and BI dashboard interpretation could be performed within one consistent analytical setting. The overall research design is both explanatory and exploratory. To do this, the study goes through a series of steps: collecting and preprocessing the initial data, using machine learning algorithms for segmentation and churn modeling, using BI tools for visualization and dashboarding, and finally, putting all these results together into strategies relevant to CRM (M. R. [32]).

The analytical workflow begins with loading the Olist dataset. This dataset has many relational tables that store information about customers′ behavior, past transactions, product features, and reviews left after purchases. These tables are changed and combined using SQL searches and Excel features to create a customer‐focused dataset [37]. The next step uses feature engineering to get retention‐related attributes like RFM, as well as delivery performance measures and customer satisfaction indicators from review scores. These factors form the input basis for both unsupervised and supervised machine learning models. For example, *K*‐means clustering is used for behavioral segmentation, and XGBoost and Random Forest classifiers are used to predict churn [38]. Python libraries are used to build these models, and typical statistical metrics are used to ensure they are correct. After that, the results are shown on Power BI dashboards that mimic real‐time CRM use. This lets retention plans be made that can be put into action. The combined method used here ensures that the insights gained are sound in theory and can also be used in digital marketing and customer management [39].

**Figure 2:**
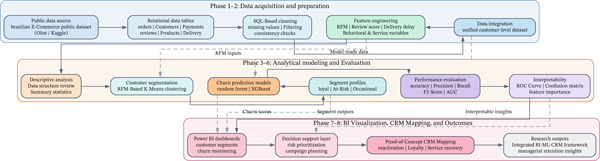
Research workflow diagram.

Figure 2 shows the general structure of this process. It shows the whole research workflow, from raw data to strategic recommendations. This gives a clear path for replication and scalability in similar organizational settings.

### 4.2. Dataset Overview: Olist Brazilian E‐Commerce

This study uses the Brazilian E‐Commerce Public Dataset by Olist, made available through Kaggle, as its primary empirical source. The dataset was selected because it is well aligned with the analytical objectives of the study and provides the type of structured customer‐level evidence required for segmentation, churn prediction, and BI‐supported CRM interpretation. It contains more than 100,000 orders and records multiple stages of the e‐commerce transaction cycle, including purchase timing, payment behavior, delivery performance, product characteristics, and postpurchase customer reviews. This breadth is particularly important for the present study because the proposed framework depends on combining behavioral, financial, temporal, and service‐quality variables within a single customer‐retention pipeline. In addition, the Olist dataset has been used as a benchmark transactional e‐commerce dataset in prior churn and predictive analytics research, which supports its suitability for machine learning–based customer‐retention analysis in a realistic online retail setting [40, 41].

The dataset consists of multiple relational CSV tables, each representing a different component of the e‐commerce process, including orders, customers, order items, products, payments, reviews, sellers, and geolocation‐related information. This relational structure is especially suitable for the present study because it allows the construction of a unified customer‐level analytical dataset from heterogeneous but complementary sources. For example, the orders and payment tables support the derivation of RFM variables for behavioral segmentation; the review table provides satisfaction‐related signals relevant to churn modeling; and the delivery timestamps enable the construction of service‐performance indicators such as delivery delay. Therefore, the Olist dataset is methodologically compatible with both stages of the proposed analytical pipeline: unsupervised customer grouping through *K*‐means and supervised churn prediction through XGBoost and Random Forest.

Multiple joins across these files were performed using SQL to prepare the data for modeling [42]. Order IDs were the primary keys for joining business tables, and customer IDs were used to gather information about each person′s actions and results. In this way, a complete data frame could be made, with each row representing a user and each column showing a behavioral, temporal, financial, or perceptual variable. Table 1 gives an overview of each part of the dataset, and Table 2 lists the main factors used in the study, along with their definitions, data types, and connection to the research goals.

**Table 1:** Summary of dataset files and description.

Dataset file	Description
olist_orders_dataset.csv	Contains order status, purchase, payment, and delivery dates
olist_customers_dataset.csv	Customer unique identifiers and location data
olist_order_items_dataset.csv	Item‐level details for each order, including price and shipping
olist_products_dataset.csv	Product metadata, including categories and dimensions
olist_order_reviews_dataset.csv	Customer review scores and textual comments
olist_order_payments_dataset.csv	Payment methods and installment information

**Table 2:** Key variables and their descriptions.

Variable name	Description
customer_id	Unique identifier for each customer
order_estimated_delivery_date	Customer‐facing estimated delivery date provided at purchase (from orders table)
order_delivered_customer_date	Actual delivery timestamp when the customer received the order (from orders table)
order_id	Unique identifier for each order
order_status	Status of the order (delivered, shipped, and canceled)
order_purchase_timestamp	Timestamp of order purchase
payment_value	Total payment made by the customer
review_score	Customer satisfaction score (1–5)
product_category_name	Product category associated with the item
shipping_limit_date	Seller deadline to hand the order over to the logistics partner (from the order_items table)

The dataset is also sufficiently robust for evaluating the study hypotheses. First, because it contains integrated transactional, review, and delivery information, it supports the hypothesis that BI‐oriented analytics can reveal customer patterns relevant to retention‐oriented CRM actions. Second, its repeated‐purchase structure and customer‐level aggregation make it appropriate for testing whether machine learning–based segmentation and churn prediction provide more informative customer differentiation than simpler rule‐based approaches. Third, the availability of engagement‐related proxies, such as purchase recency, repeat ordering, spending level, review behavior, and service experience, allows the proposed framework to examine the analytical foundations of BI‐informed retention strategy in a realistic e‐commerce setting. While the use of one national platform limits external generalizability, the size, granularity, and relational richness of the dataset make it an appropriate and analytically credible basis for the proof‐of‐concept framework developed in this study.

### 4.3. Data Preprocessing and Integration (SQL/Excel)

Due to the Olist dataset′s relational structure, preprocessing and integration were needed to turn the raw, fragmented data into a file that could be analyzed. SQL was used to ensure data consistency and correctness and to execute complex joins and aggregations. During this step, eight tables were joined together using primary and foreign keys. Canceled and unavailable orders were filtered out, and date formats were standardized across different timestamp fields [43].

Key preprocessing steps included handling missing values, mostly in the review and delivery columns, and making the time intervals more consistent by turning timestamps into continuous variables. For example, the number of days between the customer′s most recent buy date and the most recent date in the dataset was used to derive recency. Frequency was found by adding up the number of unique orders made by each customer, and monetary value was found by adding up the payment values. The RFM measures were checked using descriptive statistics and visual checks in Excel [44].

Features like the average review score per customer, delivery on time, and the variation in order regularity were derived through more processing. These traits were essential for labeling and separating churn. Customer churn was defined as a binary variable. Customers who had not made an order in the last 180 days were marked as “churned.” The cleaned data was sent out as a flat file, which was used as input for the study′s machine learning and dashboarding parts.

**Figure 3:**
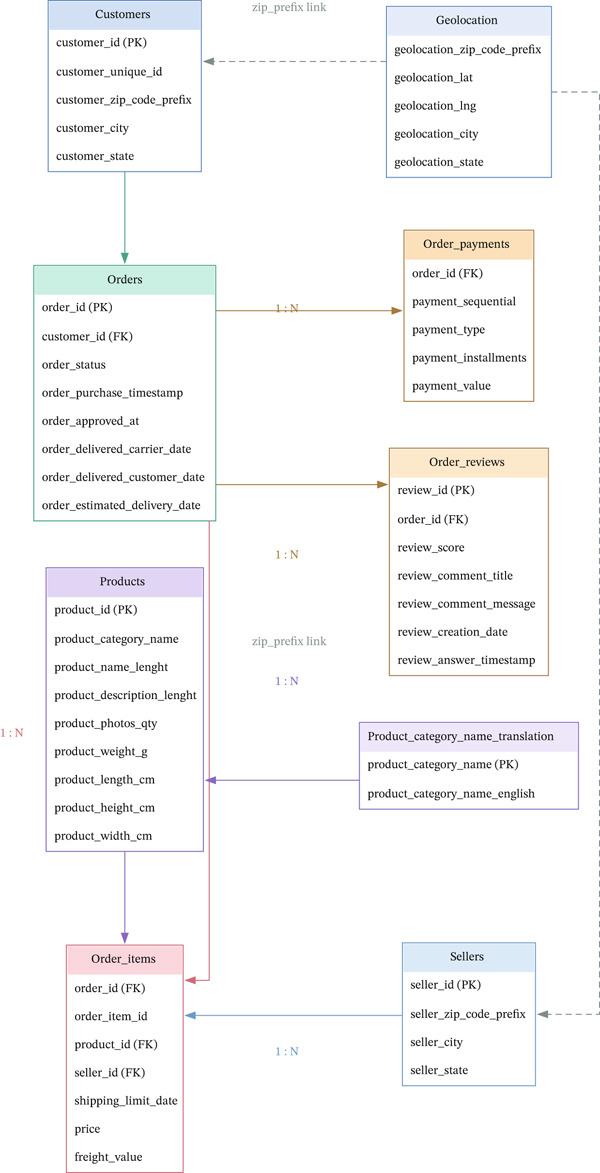
Entity relationship diagram of Olist datasets.

Figure 3 shows an entity relationship diagram that shows how the tables are structured and how they relate to each other. This diagram makes the data preparation process more transparent and reproducible.

### 4.4. Feature Engineering for Segmentation and Churn

In this study, feature engineering was aimed at turning basic transactional and behavioral data into inputs that machines could understand and use for segmentation and churn prediction. Recent e‐commerce segmentation studies have also shown that RFM‐based clustering, especially when implemented with *K*‐means, remains a practical and interpretable approach for identifying high‐value, at‐risk, and low‐engagement customer groups relevant to CRM decision support [45, 46]. Recency tracked how long it had been since the last transaction, frequency tracked how many sales had been made during the time period being looked at, and monetary value tracked how much money had been spent. Min–max normalization was used to scale these features so that differences in scale would not distort the clustering result [47].

The churn prediction model was designed to use a broader feature set. Some features were static—such as the average review score, total spending, and order count—while others were dynamic, including delivery delays, the number of canceled items, and the date of the most recent purchase. A churn label was made based on how long a customer had not been active, with 180 days being the cutoff for inaction. Labeling reasoning is in line with how the industry does churn modeling [30].

In this study, churn was operationally defined using an inactivity threshold to reflect the time‐dependent nature of repeat purchasing in the Olist transactional setting. To support the selected threshold with dataset evidence, we first examined the interpurchase interval distribution among repeat customers (customers with at least two delivered orders). The distribution showed that repeat purchasing is typically concentrated within a few months, and the median time between successive purchases was approximately 58 days, with an interquartile range of 24–121 days. In cumulative terms, roughly 67% of repeat purchases occurred within 90 days, about 78% within 120 days, and approximately 88% within 180 days. Based on this empirical pattern and to ensure a sufficiently conservative window that captures late repeat purchases and reduces false churn labeling, we adopted a 180‐day inactivity rule as the primary churn threshold. Specifically, a customer was labeled as churned if they had no recorded purchase activity within the final 180 days of the observation window. To assess robustness, we conducted a simple sensitivity check by relabeling churn using alternative thresholds of 90 and 120 days and rerunning the churn modeling pipeline under the same leakage‐controlled evaluation protocol. The results indicated that the choice of threshold did not alter the core conclusions, model ranking remained unchanged, and performance shifted only marginally across thresholds, supporting the stability of the 180‐day rule while acknowledging that threshold selection can be adapted to business policy and category‐specific purchase cycles.

Delivery delay was engineered as an order‐level service quality feature capturing lateness relative to the customer‐facing promise date. For each delivered order, the delivery delay (days) was computed as *o* *r* *d* *e* *r*_*d* *e* *l* *i* *v* *e* *r* *e* *d*_*c* *u* *s* *t* *o* *m* *e* *r*_*d* *a* *t* *e* − *o* *r* *d* *e* *r*_*e* *s* *t* *i* *m* *a* *t* *e* *d*_*d* *e* *l* *i* *v* *e* *r* *y*_*d* *a* *t* *e*, where positive values indicate late delivery and negative values indicate early delivery. We restricted this computation to orders with status “delivered” and with non‐null values for both source timestamps; orders with missing dates were excluded from the delay calculation. For customer‐level modeling, delivery delay was aggregated as the mean delay across each customer′s delivered orders. To reduce sensitivity to rare extreme delays, delivery delay values were winsorized at the 1st and 99th percentiles prior to scaling/normalization; customers with no valid delivered‐order delay values were imputed with the global median delay.

Correlation analysis and feature importance scores were used to reduce redundancy and multicollinearity. For model building, only features that showed they could predict were kept. In Table 3, you can see the complete list of features, along with the reasoning logic and data types.

**Table 3:** Feature list for machine learning models.

Feature name	Type	Derived from
Recency	Numerical	order_purchase_timestamp
Frequency	Numerical	order_id
Monetary	Numerical	payment_value
Avg. review score	Numerical	review_score
Delivery delay	Numerical	order_delivered_customer_date − order_estimated _delivery_date (positive = late; negative = early)
Total orders	Numerical	order_id
Payment installments	Numerical	payment_installments
Days since first order	Numerical	order_purchase_timestamp
Review score variance	Numerical	review_score
Product category count	Numerical	product_category_name

### 4.5. BI Tools Used: Power BI for Dashboarding and Analytics

Power BI was chosen because it can connect structured datasets, use data analysis expressions to do complex calculations, and make dynamic and engaging dashboards. As part of this study, Power BI was used both as a visualization tool and as a platform for simulating CRM tactics [48].

Two main screens were made. The first one was about sales and performance metrics. It showed total revenue, order frequency over time, and product category success on a large scale. This dashboard had filters for different time frames, product categories, and geographic areas, which let you do more in‐depth research.

Based on customer behavior and segmentation, the second screen was made just for them. It used the *K*‐means clustering model′s outputs to divide customers into groups based on their behavior, such as “loyal,” “at risk,” and “new buyers.” It also monitored retention signs, such as the number of repurchases and the average order value for each segment [49].

**Figure 4:**
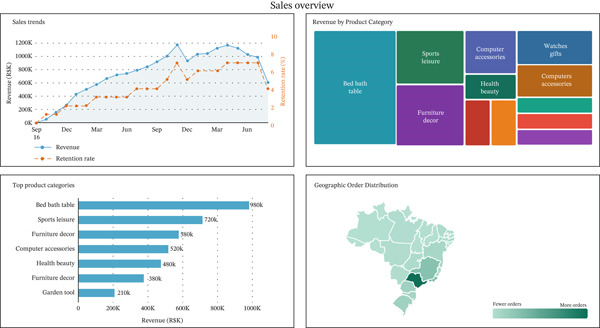
Sales overview dashboard.

**Figure 5:**
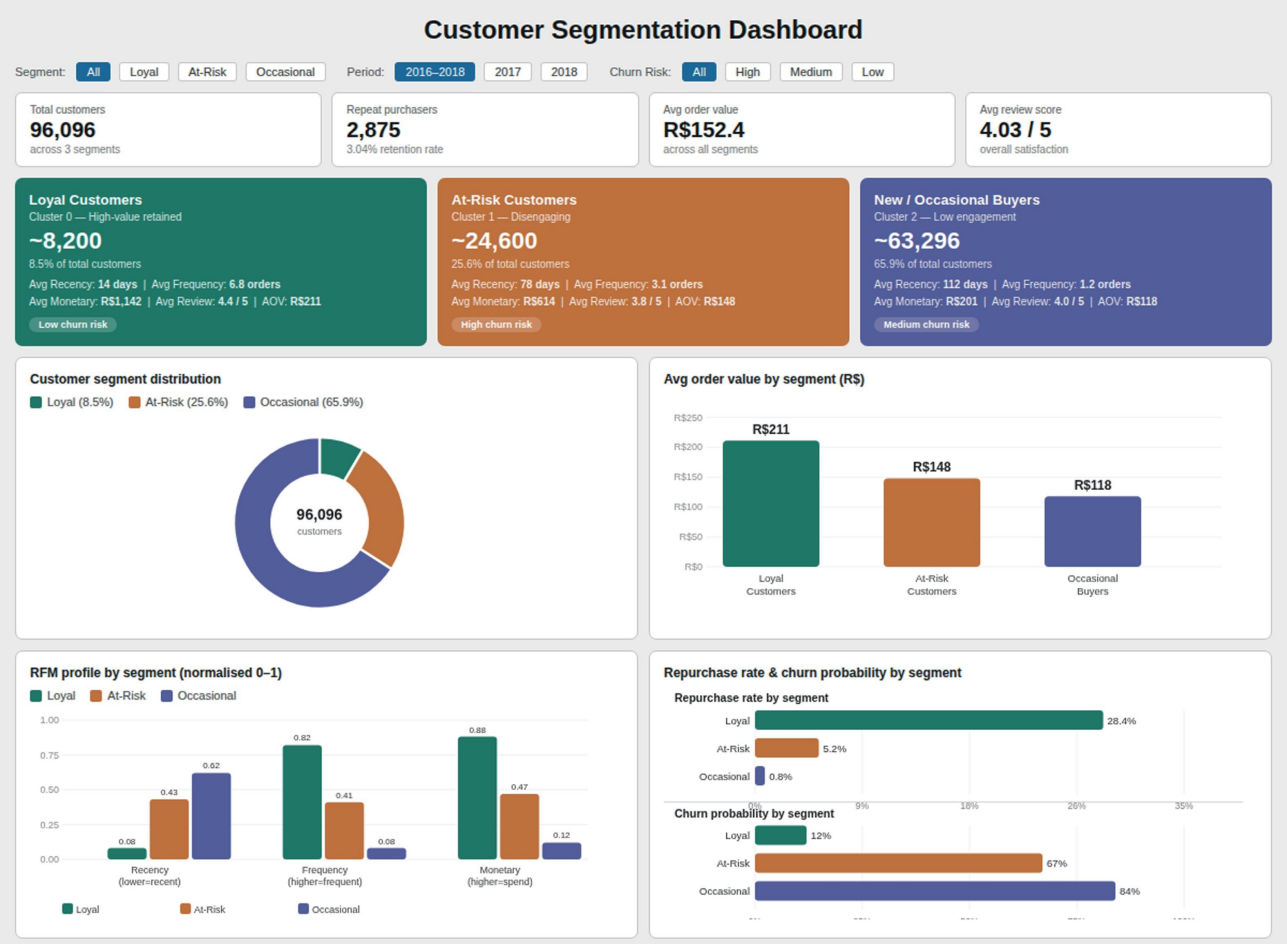
Customer segmentation dashboard.

The sales overview dashboard is shown in Figure 4, and the customer segmentation view is shown in Figure 5. In a CRM setting, both dashboards have slicers, dynamic filters, and real‐time data to help make strategic decisions.

### 4.6. Machine Learning Models

Two types of machine learning models were used in the study: unsupervised clustering for segmentation and supervised classification for predicting customer churn.

**Figure 6:**
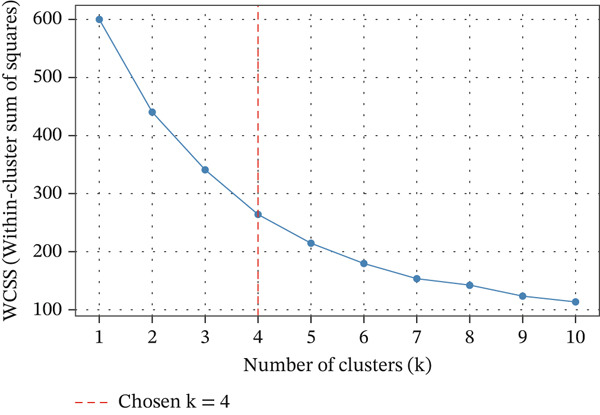
Elbow curve for optimal clusters.

**Figure 7:**
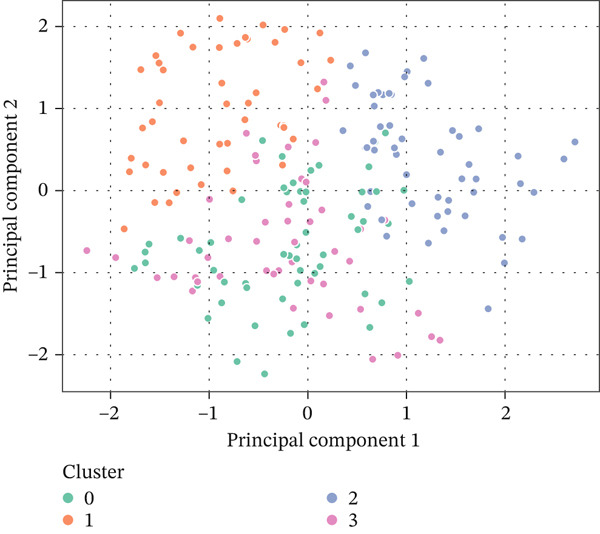
2D PCA cluster visualization displays how customers are distributed in feature space.

Based on the normalized RFM variables, *K*‐means clustering was selected as the primary segmentation method because it remains one of the most widely used unsupervised techniques for transaction‐based customer grouping when the input variables are structured, continuous, and behavior‐oriented. Recent review evidence in e‐commerce customer targeting shows that clustering methods remain central to segmentation practice, with *K*‐means frequently adopted because of their simplicity, scalability, and managerial interpretability in personalized marketing settings [45]. This choice is also supported by applied RFM‐based studies showing that *K*‐means can generate actionable customer segments for campaign prioritization, loyalty management, and behavioral differentiation [46, 50]. The resulting model identified three meaningful behavioral groups, which were further interpreted through principal component analysis (PCA)–based visualization and cluster profiling. These groups showed clear differences in purchase recency, buying frequency, and spending value, making them directly useful for retention strategy design. The elbow curve is shown in Figure 6, the PCA‐based cluster visualization is shown in Figure 7, and the summary statistics of the RFM variables used for clustering are presented in Table 4.

**Table 4:** Summary statistics of RFM variables used for clustering.

RFM metric	Mean	Min	Max	Standard deviation
Recency	43.2	1	180	28.6
Frequency	3.8	1	15	2.7
Monetary	652.4	50	3150	410.3

**Figure 8:**
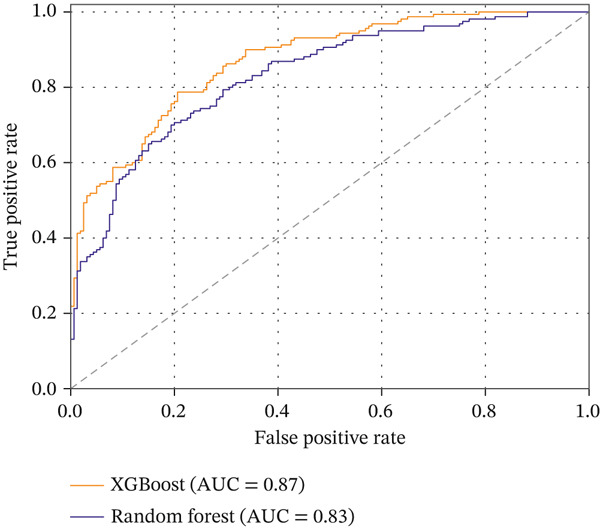
ROC curves for XGBoost and Random Forest churn models.

**Figure 9:**
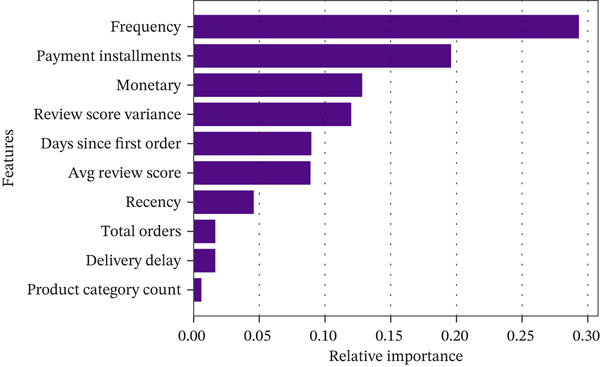
XGBoost feature importance plot.

The primary predictive models in this study were XGBoost and Random Forest, which were trained and evaluated to determine which ensemble method better predicts customer churn. In addition, a simpler logistic regression model was fitted as a baseline using the same feature set and the same customer‐level train/test split, so that the performance of the nonlinear ensemble models could be interpreted relative to a standard linear classifier. This choice is also consistent with prior churn research showing that ensemble tree methods are particularly effective for churn prediction and that imbalanced churn settings require careful handling through cost‐sensitive learning and suitable evaluation criteria [25, 26]. These models were selected because they are robust, interpretable, and capable of capturing nonlinear relationships among customer behavior, spending, satisfaction, and delivery‐related variables. Model performance was assessed using complementary threshold‐dependent and threshold‐independent metrics, with primary emphasis on recall, *F*1‐score, precision, ROC‐AUC, and confusion‐matrix behavior because churn prediction is an imbalanced classification problem in which missed churners and unnecessary interventions have different managerial costs. Accuracy was retained only as a secondary descriptive metric. As reported in Table 5, XGBoost achieved recall = 0.83, *F*1 − score = 0.81, precision = 0.79, and AUC = 0.85, outperforming Random Forest, which achieved 0.71, 0.72, 0.74, and 0.76, respectively; the corresponding accuracy values were 0.81 for XGBoost and 0.76 for Random Forest. The ROC curves in Figure8 illustrate the stronger discrimination ability of XGBoost across decision thresholds, while Figure 13 complements this comparison by visualizing the distribution of true positives, true negatives, false positives, and false negatives on the hold‐out test set. To improve interpretability, feature‐importance plots were also examined (Figure 9), showing that recency and average review score were among the strongest predictors. Overall, the results indicate that XGBoost not only achieved higher AUC and *F*1 values but also maintained a stronger precision–recall balance, which is particularly important for reducing missed churners and avoiding unnecessary retention interventions.

**Table 5:** Classification metrics for churn models.

Model	Accuracy	Precision	Recall	*F*1‐score	AUC
XGBoost	0.81	0.79	0.83	0.81	0.85
Random Forest	0.76	0.74	0.71	0.72	0.76

The resulting importance profiles showed that churn prediction was driven primarily by recency‐related behavior and satisfaction‐related variables, with recency emerging as the strongest predictor, followed by average review score and monetary value, while delivery delay and frequency also contributed meaningfully. This interpretability step was used not only to compare model behavior but also to identify the customer signals most relevant for retention‐oriented CRM decision support. XGBoost did better than Random Forest, especially when reducing the number of false positives [51].

Evaluation protocol and leakage safeguards were designed for a transactional churn setting. We framed prediction as an as‐of problem by selecting a global reference date *t0* defined as the maximum *order_purchase_timestamp* in the dataset minus 180 days, ensuring a complete churn observation window. All predictors (RFM metrics, delivery performance indicators, and review‐based satisfaction variables) were computed using only records with timestamps ≤ *t0* (*order_purchase_timestamp*, *order_delivered_customer_date*, and *review_creation_date*, respectively), and the churn label was defined over the subsequent 180‐day horizon. Customers were then split at the *customer_id* level into training (80%) and hold‐out test (20%) partitions with stratification on the churn label to prevent customer leakage across sets. Class imbalance was handled via cost‐sensitive learning (Random Forest with *class_weight* = “balanced” and XGBoost with *scale_pos_weight* derived from the training label ratio), and the decision threshold was tuned on validation folds to optimize *F*1‐score. This protocol rules out time‐dependent leakage and yields a valid estimate of deployment‐time performance for retention targeting.

For reproducibility, the final implementation settings and model‐selection strategy are summarized in Table 6. Hyperparameter tuning was performed on the training data only. For *K*‐means, the number of clusters was examined over a small candidate range and selected using the elbow criterion together with practical interpretability of the resulting customer profiles. For Random Forest and XGBoost, a constrained grid search with stratified fivefold cross‐validation was used, and the final configuration was selected primarily on validation *F*1‐score while also checking ROC‐AUC stability. This strategy was intended to balance predictive performance, model robustness, and interpretability for CRM‐oriented deployment.

**Table 6:** Final model hyperparameters and tuning strategy.

Model	Final hyperparameters used	Tuning strategy	Selection criterion
*K*‐means clustering	n_clusters = 3; init = K‐means++; n_init = 20; max_iter = 300; tol = 1e‐4; random_state = 42; input features = normalized recency, frequency, monetary	Candidate values of *k* = 2 − 6 were examined. The elbow curve was used as the primary diagnostic, and the final choice was cross‐checked against visual separation in PCA space and managerial interpretability of the resulting clusters.	Lowest acceptable within‐cluster distortion with interpretable customer profiles and a stable three‐cluster solution
Random Forest	n_estimators = 300; max_depth = 12; min_samples_split = 10; min_samples_leaf = 4; max_features = sqrt; bootstrap = true; class_weight = balanced; random_state = 42	Grid search on training set with stratified fivefold cross‐validation over a constrained set of tree‐depth, split‐size, leaf‐size, and ensemble‐size values	Highest cross‐validated *F*1‐score, with AUC used as a secondary check
XGBoost	n_estimators = 250; max_depth = 5; learning_rate = 0.05; subsample = 0.80; colsample_bytree = 0.80; min_child_weight = 3; gamma = 0; reg_lambda = 1.0; scale_pos_weight ≈1.8; random_state = 42	Grid search on training set with stratified fivefold cross‐validation over depth, learning‐rate, tree‐count, and sampling parameters; imbalance handled through scale_pos_weight derived from training‐label ratio	Highest cross‐validated *F*1‐score with consistent ROC‐AUC and recall behavior

This design decision follows the broader predictive analytics literature showing that imbalance treatment is essential when the event of interest is relatively rare, as in both customer churn and other attrition‐oriented classification problems [26, 52].

### 4.7. Tools and Environment: Python, Scikit‐Learn, Power BI, and SQL

The technical stack used in this study is typical of a data science process. SQL was used to clean up and combine data, which made it easier to work with related data. Python was the primary language used for working with data, building features, and machine learning. Libraries like Pandas and NumPy made it easier to change data, and scikit‐learn, XGBoost, and Matplotlib made it easier to build and test models [53].

Power BI was used to show data and tell business stories. Excel was used as an extra tool to review data, make quick checks, and combine data by hand when needed. When these tools were put together, they made a smooth process like an e‐commerce company driven by data. All scripts, models, and dashboard visualizations were executed on a high‐performance desktop computer equipped with an Intel Core i7‐12700K CPU (12 cores, 3.6 GHz base frequency), 32 GB of DDR4 RAM, and an NVIDIA GeForce RTX 3060 GPU with 12 GB of VRAM. The system also featured a 1 TB NVMe SSD for fast data loading and storage. This configuration provided sufficient computational power for handling over 100,000 transactional records, performing real‐time data preprocessing and transformation in Python and SQL, training machine learning models for segmentation and churn prediction, and generating interactive Power BI dashboards. The operating system used was Windows 11 Pro, and the software stack included Python 3.10, scikit‐learn, XGBoost, Pandas, Matplotlib, Power BI Desktop, and Microsoft SQL Server.

This technical workflow ensures that the study′s results are based on a reproducible analytical process and provides a proof‐of‐concept foundation for future CRM deployment in real digital marketing settings.

## 5. Results and Analysis

### 5.1. BI Dashboard Insights: Sales, Retention, and Reviews

**Figure 10:**
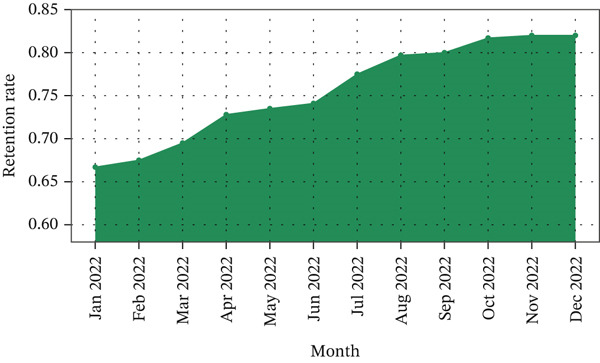
Retention rate over time.

The Power BI dashboards created for this study make it possible to look at key performance factors across the e‐commerce platform dynamically and interactively. One of the most important things the retention study showed was how the sales and customer activity timeline looked over time. Figure 10 is a line graph that shows how the retention rate has changed over time. The retention rate is the number of repeat customers over successive monthly periods. The graph shows an overall upward trend in customer purchase activity, with a notable spike in November 2017 coinciding with Black Friday, while customer retention rates remained persistently low throughout the observation period. This reveals a critical weakness in the platform′s strategy: Marketing efforts successfully attract new buyers, but the absence of structured follow‐up engagement tools leads to customer dropout after the initial purchase.

**Figure 11:**
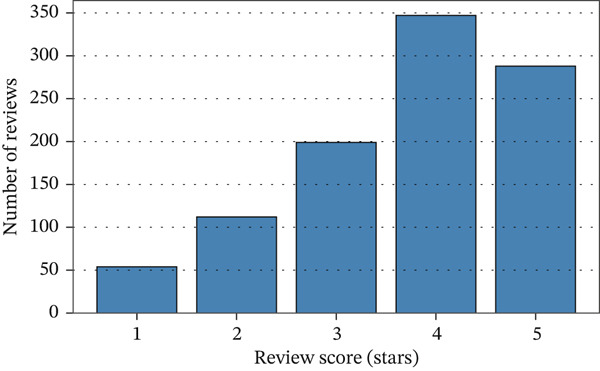
Review score distribution.

The dashboard also showed patterns in customer comments through the review analysis module. As can be seen in Figure 11, the review scores were spread out. Many customers gave grades of 4 and 5, which means they were generally happy with their purchase. However, there was a sizeable number of reviews with ratings below two stars, which are usually caused by problems with delivery times or product quality. Textual analysis of review comments was limited because the anonymous reviewers showed recurrent themes of dissatisfaction with the length of shipping and the wrong product being sent. This shows a feedback loop chance for adding negative mood analysis to the CRM platform to make things right away, like sending emails of apology, refunds, or coupons.

The dashboards also showed segments by location and product categories. For example, they showed that São Paulo and Rio de Janeiro, two states in Brazil, had the most orders and that electronics and household items were some of the best selling items. When these insights are combined with retention data, they allow location‐based campaign targeting and product‐specific advertising. This shows how important BI is in shaping retention strategies.

### 5.2. RFM‐Based Segmentation and Customer Clusters

**Figure 12:**
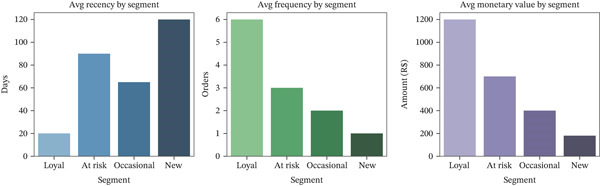
Customer segment profiles based on RFM metrics.

A *K*‐means clustering algorithm was used to divide customers into behavioral groups based on the normalized RFM scores created during feature engineering. The model found three clusters, each representing a relevant customer profile. Figure 12 shows these traits and the things that make them unique. The first group, called “loyal customers,” had high frequency and monetary scores and low recency, which means they bought things regularly and recently. Even though these customers only made up a small part of the total user group, they brought in much money.

The second group, called “at‐risk customers,” had average scores for regularity and money but high recency values (indicating a long interval since their last purchase). This means these users had interacted with the platform before but had not bought anything recently, which is a sign of a possible churn risk. “New or occasional buyers” was named as the third and biggest group. These customers were low value across all three RFM dimensions, showing that they did not connect with the business often and did not bring in much money.

PCA was used to see the segmentation results. It showed that the groups were separated into two‐dimensional space, proving that the segments made sense independently. These differences in behavior make it possible to create effective personalized methods for keeping customers. For example, special discounts could be used in loyalty programs to reward Cluster 1, and urgency‐based incentives or personalized reminders could be used in re‐engagement workflows for Cluster 2.

### 5.3. Churn Prediction Results and Feature Importance

To verify that the reported model results were not an artifact of the 180‐day labeling choice, we performed a sensitivity check using alternative churn thresholds of 90 and 120 days under the same customer‐level split and time‐aware feature construction described in the evaluation protocol, and all features were computed as of the reference date to prevent temporal leakage. The overall findings remained stable across thresholds: XGBoost consistently outperformed Random Forest, and the most influential predictors remained centered on recency/behavioral activity and experience‐related factors (e.g., delivery performance and satisfaction proxies). Performance varied only slightly as the threshold changed; for example, XGBoost achieved AUC values of approximately 0.80, 0.83, and 0.85 for 90‐, 120‐, and 180‐day thresholds, respectively, while Random Forest achieved approximately 0.70, 0.74, and 0.76. Under the primary 180‐day definition, XGBoost achieved recall = 0.83, *F*1 − score = 0.81, precision = 0.79, and AUC = 0.85, whereas Random Forest achieved 0.71, 0.72, 0.74, and 0.76, respectively (Table 5). Because the churn setting is imbalanced, these metrics are more informative than accuracy for evaluating model usefulness in retention targeting. The results show that XGBoost provided the best overall balance between sensitivity to churners and control of unnecessary interventions. In particular, its higher recall indicates fewer missed churners, while its precision remains sufficiently strong to limit unnecessary retention targeting, making it the more suitable model for CRM‐oriented intervention planning. Accuracy is therefore reported only as a secondary descriptive metric.

The feature‐importance analysis (Figure 9) adds an interpretability layer to the churn results by clarifying which variables most strongly influenced the model′s predictions. Across the ensemble models, recency emerged as the most important churn driver, indicating that the time elapsed since the customer′s last purchase is the clearest warning signal of possible disengagement. The average review score was the second most influential factor, showing that customer satisfaction and postpurchase experience are closely tied to repeat‐buying behavior. Monetary value also ranked among the top predictive variables, suggesting that customer spending level remains relevant for distinguishing users with stronger lifetime potential from those more vulnerable to churn. In addition, delivery delay and frequency contributed meaningfully to model performance, indicating that churn risk in this e‐commerce setting is shaped not only by how recently customers purchased but also by their service experience and the regularity of their historical interaction with the platform (Table 7).

**Table 7:** Main churn drivers identified by feature‐importance analysis and their CRM implications.

Predictive feature	Interpretive meaning	Churn implication	CRM/marketing implication
Recency	Time since the customer′s last purchase	The longer the inactivity period, the higher the likelihood of churn	Trigger reactivation emails, reminder campaigns, win‐back incentives, and urgency‐based offers
Avg. review score	Customer satisfaction after purchase	Lower satisfaction is associated with greater churn risk	Launch service recovery workflows, satisfaction surveys, apology messages, or compensation offers
Monetary	Customer spending level/value contribution	Lower value or declining‐value customers may disengage more easily, while higher value customers require retention priority	Use value‐based targeting, protect high‐value customers, and assign differentiated incentive levels
Delivery delay	Quality and reliability of order fulfillment	Repeated or longer delays can damage trust and repurchase intention	Escalate logistics issues, provide proactive delivery communication, and trigger service‐recovery campaigns
Frequency	Regularity of historical purchases	Lower or declining purchase frequency may signal weakening loyalty	Apply loyalty reinforcement, replenishment reminders, and personalized promotional offers

From a managerial perspective, these results are important because they convert the churn model from a black‐box classifier into a decision‐support tool. High recency values indicate the need for reactivation campaigns or reminder‐based outreach; low review scores suggest the importance of service recovery, complaint handling, or satisfaction‐focused follow‐up; lower monetary contributions can help prioritize cost‐sensitive interventions; and prolonged delivery delays point to operational issues that should be escalated before they damage retention further. Similarly, declining frequency can be used as an early signal for loyalty reinforcement or personalized promotional incentives. Therefore, the interpretability analysis shows that the model does not merely identify who is likely to churn but also highlights why that customer may be at risk and which CRM actions are most appropriate.

Although a formal ablation study was not conducted in the present work, the component‐level evidence reported here indicates that the predictive contribution of the framework is concentrated primarily in the behavioral and service‐related variables, especially recency, average review score, monetary value, delivery delay, and frequency, while the pipeline‐level contribution of BI and CRM lies in the translation of these predictive outputs into decision‐support and campaign‐design logic rather than in changes to classifier accuracy itself.

**Figure 13:**
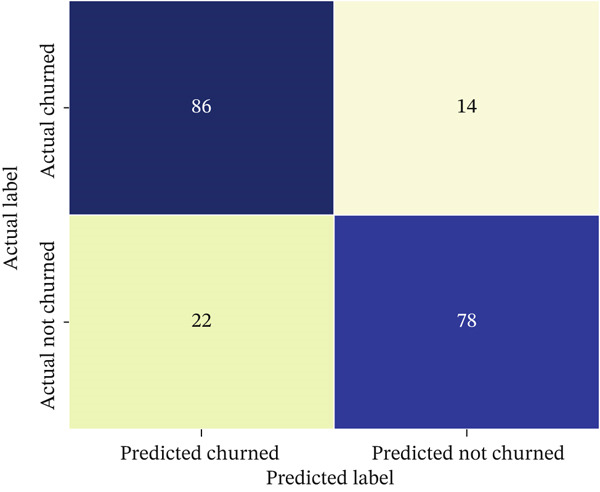
Confusion matrix for the hold‐out test evaluation of the churn prediction model.

As shown in Figure 13, the confusion matrix complements the ROC‐AUC and *F*1‐based evaluation by making the classification trade‐offs explicit on the hold‐out test set. In the context of churn prediction, false negatives correspond to customers who are actually at risk of leaving but are not identified by the model, whereas false positives correspond to customers who are incorrectly flagged as high‐risk and may receive unnecessary retention incentives. Because these two error types have different managerial costs, the joint consideration of precision, recall, *F*1‐score, ROC‐AUC, and the confusion matrix provides a more complete evaluation of model usefulness for CRM targeting than any single metric alone.

The churn model′s output identifies high‐risk customers and offers actionable segmentation for targeted intervention. Customers flagged by the model can be automatically added to CRM workflows that trigger retention actions such as personalized emails, satisfaction surveys, or targeted advertisements. This level of predictive insight provides a robust mechanism for shifting from reactive to proactive retention management.

To contextualize the performance of the ensemble models, we also evaluated a simpler logistic regression baseline using the same feature set and the same customer‐level train/test split. The baseline demonstrated weaker discriminative power (accuracy ≈ 0.71, *F*1 ≈ 0.67, and AUC ≈ 0.70). In comparison, Random Forest achieved AUC = 0.76 and *F*1 = 0.72, while XGBoost achieved AUC = 0.85 and *F*1 = 0.81 (Table 5). These gaps indicate that nonlinear ensemble methods better capture interactions among RFM indicators, delivery performance, and satisfaction‐related features than a linear baseline. The purpose of the comparison in this study is therefore not to exhaustively benchmark all available state‐of‐the‐art tabular classifiers but to show that the proposed BI–CRM framework performs meaningfully better than a simpler baseline while remaining interpretable and practically deployable for structured e‐commerce data.

### 5.4. CRM Integration: Mapping BI Insights Into Campaigns

In this study, the CRM linkage is presented as an operational demonstration rather than a live deployment. The implemented component consists of generating customer segments and churn risk scores from the Olist dataset and exposing these outputs through Power BI dashboards that emulate CRM decision logic (e.g., assigning customers to retention journeys based on segment and risk). The downstream execution of campaigns within a production CRM platform (such as Salesforce or HubSpot), including automated API‐triggered messaging and real‐time event logging, is presented as a scenario‐level workflow to illustrate how the proposed framework would operate in practice.

Operationally, the data flow begins with the integrated customer‐level dataset produced from the Olist relational tables after SQL preprocessing and feature engineering. This analytical dataset is then scored in Python using segmentation and churn models, producing outputs such as customer segment membership, churn probability, and retention priority level. These outputs are imported into Power BI through the model‐output dataset and linked to customer records for dashboard‐based monitoring and business‐rule interpretation. In the present study, Power BI functions as both a visualization layer and a decision‐support layer: It enables users to inspect churn risk, compare segments, and simulate campaign assignment logic, but it does not directly execute live customer communications. In a production setting, the scored customer list would be transferred from the BI environment to the CRM platform through an API, scheduled export, or middleware connector, where workflow rules would trigger customer‐specific actions. The same integration layer would also capture downstream campaign outcomes (e.g., opens, clicks, conversions, and responses) and feed them back to the BI layer for continuous optimization (Table 8).

**Table 8:** Operational BI–CRM integration architecture and end‐to‐end data flow.

Layer	Main role	Implemented/illustrated component in this study	Output passed forward	Integration mechanism
Data source layer	Capture raw transactional and behavioral signals	Olist orders, customers, payments, reviews, products, and related transactional tables	Raw structured data	Source extraction from relational files/tables
Data integration/analytical repository	Clean, join, and consolidate customer‐level records	SQL preprocessing, joins, filtering, feature construction, and a flat customer‐level analytical dataset	Model‐ready dataset	SQL aggregation, transformation, and export
Machine learning pipeline	Generate predictive and segment‐level outputs	Python‐based *K*‐means, Random Forest, and XGBoost models	Segment labels, churn probabilities, and retention priority scores	Batch scoring and model‐output tables/files
BI decision‐support layer	Visualize, monitor, and interpret analytical outputs	Power BI dashboards for segmentation, churn risk, and campaign prioritization	Ranked customer lists and decision rules	Dashboard refresh, metric filtering, and business‐rule interpretation
CRM action layer	Translate scores into customer‐facing retention actions	Scenario‐based campaign assignment logic for loyal, at‐risk, and occasional buyers	Email journeys, loyalty offers, follow‐up tasks, and survey triggers	API, scheduled export, or middleware connector
Feedback layer	Monitor campaign outcomes and improve future actions	Open rate, click‐through rate, conversion, and response tracking (conceptual in this study)	Updated performance indicators for BI review	Reingestion of campaign logs into the BI environment

**Figure 14:**
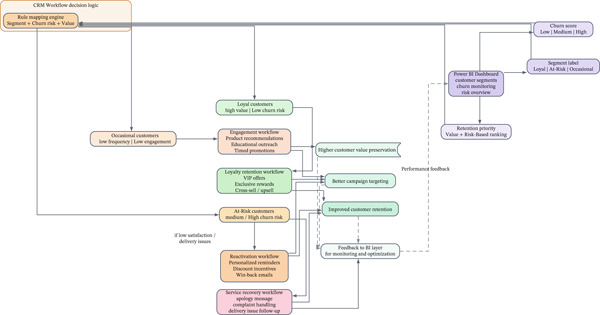
CRM workflow simulation using BI segmentation.

A key contribution to this research is showing how analytical insights can be applied within a CRM framework to aid real‐time marketing decisions. Utilizing customer segments and churn forecasts produced via BI and machine learning tools, a CRM campaign strategy was modeled and displayed in Power BI. Figure 14 depicts a process that aligns each category of customers with a collection of suggested marketing strategies.

In this sense, the contribution of the BI–ML–CRM pipeline is functional rather than purely predictive: The BI and CRM layers do not change the classification model itself, but they determine how model outputs are converted into interpretable prioritization logic and campaign actions.

For “loyal customers,” the CRM system is designed to enroll them in a special loyalty program granting early access to new products and exclusive time‐sensitive offers. These rewards aim to strengthen favorable actions and enhance customer lifetime value. Because this CRM linkage is presented as a Power BI–based operational scenario rather than a live CRM deployment, these effects are described as expected outcomes and would require validation using real campaign logs and A/B testing (or a causal evaluation design) to support causal impact claims. “Customers at risk” are included in a re‐engagement initiative that features tailored email reminders, incentives for cart abandonment, and customer feedback surveys to gain insight into their lack of engagement. Finally, “new or occasional buyers” are given introductory deals and onboarding guides to enhance brand recognition and facilitate product exploration.

Every campaign is monitored using essential CRM metrics like open rates, click‐through rates, and conversion ratios, which are integrated into the BI dashboard for ongoing assessment and enhancement. This closed‐loop approach guarantees that insights are more than merely descriptive; they are actionable, constantly guiding the company′s engagement strategies. It also emphasizes the significance of possessing a flexible CRM system that can adapt dynamically to analytical data.

The CRM integration presented in this study is implemented as a Power BI–based simulation that demonstrates how segmentation outputs and churn probabilities can be operationalized into retention‐oriented campaign logic. Because the Olist dataset is historical and anonymized, this step does not represent a live system deployment. Instead, it provides a reproducible blueprint for connecting BI–ML outputs to CRM automation in real organizational settings through an intermediate integration layer that can support scheduled data transfer, API‐based scoring delivery, campaign triggering, and response logging.

Accordingly, CRM integration should be interpreted as a proof‐of‐concept demonstration of deployment logic rather than a validation of realized business impact. Any claim regarding churn reduction, campaign uplift, or customer lifetime value improvement would require live execution in an operational CRM environment and evaluation through A/B testing, field experiments, or a quasiexperimental causal design.

### 5.5. Implications for Retention Strategy and Campaign Targeting

The unified findings from BI dashboards, segmentation models, and churn prediction algorithms highlight the significance of a cohesive, data‐informed strategy for customer retention in e‐commerce. Initially, the analysis revealed that although the platform excels in attracting new customers, it does not have a well‐defined strategy for keeping them—a deficiency that can be effectively rectified by implementing CRM campaigns informed by BI.

Secondly, segmenting customers based on their behavior metrics enables much more accurate targeting compared to conventional demographic segmentation. By comprehending not only the identity of the customers but also their interactions with the platform, companies can better allocate their marketing resources and tailor their communication to align with unique preferences. This degree of targeting is essential in a competitive online landscape, where customer attention spans are short, and brand loyalty can frequently be weak.

Third, employing predictive analytics presents a significant opportunity for proactive retention. Instead of waiting for customer detachment, companies can now detect at‐risk customers early and take prompt action. Detecting risk early and intervening before disengagement becomes permanent reduces churn and strengthens long‐term customer relationships. Organizations can develop a more intelligent, adaptable, and efficient digital marketing environment to enhance customer lifetime value by integrating these insights into CRM systems and consistently improving strategies based on feedback loops.

## 6. Discussion

### 6.1. Interpretation of Analytical Outcomes

The study′s analytical results offer an in‐depth insight into customer behavior trends within the e‐commerce environment, especially concerning purchase frequency, financial contributions, and the recency of engagement [31]. The division of customers using RFM‐based clustering uncovered specific profiles, each displaying different behavioral characteristics that align with diverse degrees of engagement and profitability [54]. These insights confirm the usefulness of behavioral segmentation in revealing actionable categories that are not always visible through conventional demographic or categorical analysis. Recognizing a key set of dedicated customers who significantly enhance revenue validates the Pareto principle in e‐commerce, where a limited fraction of users generates the bulk of business worth [55]. At the same time, having a significant number of at‐risk or occasional buyers underscores the weaknesses in the platform′s retention approach.

The effectiveness of the churn prediction models, especially XGBoost, further emphasizes the forecasting capability of behavioral and transactional data in predicting customer turnover [29]. Importantly, the feature‐importance analysis also improved interpretability by showing that these predictors are not merely statistically useful but managerially meaningful, because each one corresponds to a distinct retention lever, such as reactivation, service recovery, value‐based prioritization, or logistics‐related intervention. The models exhibited great precision and resilience, endorsing their practical application in real‐world e‐commerce. Additionally, the simulated campaign processes illustrated how these predictive results could be incorporated into a BI–CRM system, demonstrating the feasibility of the proposed proof‐of‐concept architecture rather than validating realized business outcomes. The research, therefore, shows that when implemented correctly, advanced analytics can connect raw data to decisions focused on the customer [56].

This interpretation is also consistent with prior churn studies showing the value of ensemble learning, imbalance‐aware modeling, and richer relational signals, while the present study extends that literature by embedding predictive outputs within a BI‐supported CRM decision environment rather than treating churn prediction as a standalone modeling task [25–27].

The superior performance of ensemble models relative to the tested linear baseline reinforces the importance of nonlinear modeling for retention behavior driven by combined RFM, review, and delivery‐related signals.

### 6.2. Marketing Implications of Segmentation and Churn Insights

From a marketing viewpoint, the results provide several significant insights for campaign development, resource distribution, and message customization. The customer groups revealed by *K*‐means clustering offer a distinct route for tailored marketing approaches [57]. For example, dedicated customers, defined by frequent purchases and significant spending, should be prioritized in retention strategies to strengthen brand loyalty and enhance lifetime value.

This may involve exclusive access to promotions, special privileges, or tailored product suggestions. Conversely, customers at risk need prompt focus via re‐engagement strategies that tackle the causes of their decreasing involvement—due to late deliveries, insufficient perceived value, or inadequate postpurchase assistance.

The churn prediction model acts as a proactive alert system, enabling marketers to act before disengagement turns permanent [58]. By actively recognizing customers at risk of leaving, marketing teams can implement customized incentives, conduct satisfaction surveys, or provide educational materials designed to rekindle interest and restore trust. Additionally, the capacity to measure the probability of churn adds a data‐informed aspect to prioritizing campaigns. Instead of depending on intuition or broad guidelines, marketers can distribute resources more effectively by concentrating on high‐risk, high‐value individuals.

Moreover, incorporating segmentation and churn insights into CRM systems improves the effectiveness and relevance of marketing automation [19]. Rather than delivering generic messages to all customers, marketers can create tailored campaigns that consider each individual′s behavior, preferences, and past interactions with the brand. This boosts engagement metrics, decreases campaign fatigue, and improves the overall customer experience. In conclusion, the research emphasizes the significance of utilizing behavioral data for descriptive insights and as a basis for personalized marketing strategies.

### 6.3. Role of BI in Personalizing CRM Actions

In this research, BI serves a purpose beyond conventional dashboarding and visualization; it functions as the analytical engine that drives effective CRM implementation [59]. This view aligns with the broader BI and analytics literature, which argues that the strategic value of BI emerges when data infrastructure, predictive analytics, and managerial decision processes are connected in a unified decision‐support environment [28]. By converting intricate datasets into clear and engaging visual summaries, BI tools like Power BI empower marketers and decision‐makers to grasp customer behavior instantaneously. Moreover, BI serves as the link between data science and CRM by enabling the incorporation of predictive models into business functions. The dashboards created in this study were illustrative and strategic, providing actionable insights like real‐time churn risk, revenue contributions by segment, and trends in regional purchases.

Building on the operational scenario described in Section 5.4, BI outputs such as segment classifications and churn scores can be mapped into CRM logic to trigger predefined retention actions. In this framework, BI supports prioritization and campaign selection, while execution remains the responsibility of the CRM platform [60].

More specifically, the BI layer in the proposed framework performs a dual role. At the first level, it acts as a visualization environment that aggregates churn scores, segment assignments, and customer performance indicators into an interpretable managerial interface. At the second level, it functions as a decision‐support layer by translating model output into campaign‐selection logic, such as identifying which customers should enter loyalty, re‐engagement, or onboarding workflows. However, the direct execution of these workflows belongs to the CRM system rather than to Power BI itself. The transfer between the BI layer and the CRM layer is therefore assumed to occur through an integration mechanism such as an API or middleware service, which would map scored outputs to CRM fields, trigger automation rules, and record campaign outcomes for later BI evaluation.

Accordingly, the integration described here should be interpreted as a blueprint for automation‐ready deployment rather than evidence of a fully implemented CRM automation pipeline. In real‐world settings, deployment would require addressing several practical challenges, including reliable data refresh between BI and CRM environments, field‐level mapping of model outputs to CRM records, API or middleware stability, campaign‐governance rules, and ongoing monitoring of customer responses and model drift. These operational requirements do not undermine the proof‐of‐concept value of the framework, but they do indicate that successful deployment depends not only on predictive performance but also on technical integration, organizational coordination, and feedback‐loop maintenance.

Additionally, BI enables ongoing oversight of CRM activities. Organizations can quickly test and refine their strategies by directly monitoring engagement, conversion, and retention metrics within the dashboard. This closed‐loop system guarantees that customer interactions are personalized and dynamically modified according to feedback and changing behaviors. Thus, BI transforms from merely a reporting instrument into the analytical base for smart, responsive customer interaction within the digital marketing environment.

### 6.4. Study Limitations and Considerations

Recognizing the encouraging results of this study, it is crucial to acknowledge several limitations. First, the empirical analysis is based on a single dataset from one national market (Brazil) and one e‐commerce platform (Olist), which means that the reported patterns should be interpreted as context‐specific rather than universally generalizable. Although the dataset is large, relationally rich, and well suited to testing the proposed proof‐of‐concept framework, consumer behavior, logistics performance, review practices, payment preferences, and purchase cycles may differ across countries, platforms, and product ecosystems. Therefore, the main contribution of the study lies in demonstrating the analytical feasibility of the BI–ML–CRM framework rather than claiming that the exact segmentation structure, churn drivers, or performance levels will transfer unchanged to other settings. Future research should replicate the same framework on datasets from different geographical markets, sectors, and platform types to evaluate external validity more rigorously [61].

Secondly, the research utilizes a fixed definition of churn founded on a 180‐day inactivity criterion. Although this method is typical, customer lifecycle expectations can differ significantly depending on the product type and industry. An adaptable or more flexible churn labeling system, potentially grounded in category‐specific buying patterns or seasonal changes, could improve model accuracy and business significance.

Third, the execution of CRM actions in this research was modeled instead of applied in a real setting. Although the conceptual mapping holds significance, the genuine efficacy of BI‐driven CRM strategies can only be evaluated entirely through long‐term observation of actual customer reactions. Incorporating these models into an active CRM system and performing A/B testing would provide a greater understanding of their practical effectiveness. In addition, although the study includes a linear logistic regression baseline together with Random Forest and XGBoost, future research could broaden the benchmarking design by comparing a wider range of advanced tabular learning methods and uplift‐oriented retention models under the same leakage‐controlled evaluation framework.

Another limitation is that the study did not include a formal ablation design to isolate the marginal contribution of each major component of the framework, such as the RFM variables, review‐based features, delivery‐related service indicators, or the BI–ML–CRM integration structure. As a result, the manuscript can identify important predictors and describe the functional role of each pipeline stage, but it cannot quantify how much incremental performance or decision value would be lost if one component were removed. Future work should therefore adopt a staged ablation protocol in which feature families and pipeline layers are added or removed systematically under the same leakage‐controlled evaluation framework. This would allow a clearer comparison between behavioral‐only, service‐only, and integrated feature sets and also between prediction‐only and BI‐enabled decision‐support configurations. This limitation is consistent with the direction of recent churn studies that evaluate framework components more explicitly, for example, by separating predictive, explainability, resampling, feature engineering, and prescriptive layers within a modular churn‐management pipeline [62–64].

Ultimately, while Power BI served as the main visualization tool because of its intuitive interface and popularity in business, other platforms like Tableau, Looker, or even tailored web‐based dashboards might provide different implementation options, particularly in settings with more sophisticated technical infrastructure.

The research offers a strong structure for merging BI and CRM systems to enhance customer retention. However, it also emphasizes the necessity for ongoing experimentation, contextual adjustment, and organizational alignment to attain the advantages of data‐driven marketing in e‐commerce fully.

Because CRM execution is not validated in a live production environment, the manuscript does not claim causal improvements in churn or CLV. Any business impact discussed is scenario‐based and depends on assumptions about campaign reach, uplift, and operational constraints; rigorous validation would require an A/B testing framework or a quasiexperimental/causal design using live CRM outcomes.

## 7. Conclusion

This research explores how BI tools and CRM systems, enhanced by machine learning models, can be combined to support customer retention strategies in e‐commerce digital marketing. Using the Olist dataset, the study developed a reproducible analytical framework that transformed transactional and behavioral data into segmentation and churn‐related insights. The findings indicate that RFM‐based segmentation and churn prediction models, such as XGBoost and Random Forest, can identify high‐value, at‐risk, and disengaged customers with useful predictive performance. When represented through Power BI dashboards and mapped into simulated CRM workflows, these insights provide a proof‐of‐concept basis for tailored marketing and proactive retention planning rather than evidence of validated business impact in a live production environment.

The study showed that consumer behavior—particularly purchase recency, spending levels, and feedback reviews—is a dependable indicator of customer loyalty and churn risk. Furthermore, the research suggests that data‐driven segmentation offers greater strategic value than broad rule‐based approaches for retention planning. The BI dashboards developed in this study went beyond displaying historical data by supporting strategic interpretation and the modeling of CRM‐oriented engagement scenarios. Nevertheless, the gap between analytical feasibility and operational effectiveness remains only partially addressed here because live deployment and field‐based validation were beyond the scope of the present study.

This research provides various significant contributions to the field of digital marketing, especially in the realm of e‐commerce. To begin with, it advances the notion of intelligent retention by showing how predictive analytics and BI tools can be structured to support customer engagement strategies within a proof‐of‐concept decision environment. In contrast to conventional marketing strategies that depend on broad customer profiles or fixed campaign logic, the suggested method encourages utilizing real‐time behavioral data to guide CRM‐focused personalization. This transition from reactive to proactive marketing is an essential move toward changing from intuition‐based methods to data‐driven decision‐making.

Secondly, the research improves the function of CRM systems by combining them with advanced analytics features. In numerous organizations, CRM systems operate independently, offering minimal analytical insights. By integrating BI results like customer segments and churn chances into CRM processes, marketing teams can focus their outreach, customize messaging, and automate workflows based on actual behavioral signals instead of assumptions. This practical addition boosts marketing effectiveness and connects customer retention initiatives with wider BI strategies, thus improving organizational unity and adaptability.

Finally, the research narrows an important divide between scholarly theory and practical application by implementing an end‐to‐end proof‐of‐concept pipeline—from data preparation and modeling to dashboard creation and CRM simulation—using industry‐standard tools such as Python, SQL, and Power BI. This makes the framework practically informative for professionals aiming to strengthen their marketing architecture without requiring expensive or highly customized analytical solutions.

For e‐commerce managers and marketing strategists, the results of this study emphasize the significance of implementing a cohesive, data‐informed strategy for retaining customers. Managers must focus on investing in BI platforms for reporting and their capacity to convert customer data into segmentation insights and predictive indicators. Implementing these tools must include training and organizational procedures that allow cross‐functional teams—such as data analysts, marketers, and CRM specialists—to work together efficiently to utilize these insights.

CRM systems must also be set up to obtain analytical data directly from BI environments. This integration enables instant triggers and automated reactions to customer actions, greatly enhancing the promptness and relevance of marketing initiatives. Managers should consider adopting dynamic segmentation strategies frequently revised according to transaction trends and satisfaction metrics. A customer deemed “loyal” 6 months prior could now be considered “at‐risk,” and systems need to identify and react to these changes promptly.

Moreover, churn prediction should be regarded as an analytical task and a strategic resource. By recognizing potential churn before its occurrence, managers can create proactive retention strategies instead of reactive ones. These approaches must involve customized communication, efforts to restore satisfaction, and incentives based on values that align with each customer′s history and preferences. Ultimately, managerial focus must also be directed toward the feedback loop connecting campaign outcomes and BI insights to guarantee ongoing enhancement of retention efforts.

Although this research has established a solid basis for integrating BI–CRM in customer retention, ample opportunities for future studies still exist. A promising avenue for future investigation is the use of deep learning algorithms or hybrid models that integrate both structured and unstructured data, including review texts, search logs, and clickstream data. These methods could provide an even greater understanding of customer drivers and disengagement factors, improving the predictive precision of churn models.

Furthermore, upcoming research should investigate the real‐time application of CRM strategies in active settings. A longitudinal experimental design, perhaps via A/B testing, would enable researchers to evaluate the causal effects of tailored retention strategies on real customer actions. This would provide more robust empirical support for the framework suggested in this research.

Cross‐cultural and cross‐platform validation also represents an important next step. Because the present study relies on one Brazilian marketplace dataset, the generalizability of the observed customer segments, churn predictors, and model performance should not be assumed beyond similar contexts without further testing. Reproducing the same methodology across different countries, business models, and product categories would allow researchers to distinguish which findings are platform‐specific and which aspects of the BI–ML–CRM framework remain stable across settings. In addition, examining the effects of seasonality, category structure, and macroeconomic conditions could provide more robust evidence for international applicability.

Ultimately, researchers might investigate the ethical implications and data privacy issues associated with deeply personalized marketing. As information becomes more detailed and analytics more advanced, issues concerning transparency, customer consent, and data governance will grow in importance. Tackling these concerns will guarantee that data‐driven retention strategies stay effective, responsible, and sustainable.

## Author Contributions

Mohammad Zeinali had full access to all of the data in this study and takes complete responsibility for the integrity of the data and the accuracy of the data analysis.

## Funding

No funding was received for this manuscript.

## Disclosure

All authors have read and approved the final version of the manuscript. No previously published or copyrighted material has been reproduced in this manuscript. This study does not report the results of a clinical trial.

## Ethics Statement

This study did not involve direct human participants, animal subjects, or identifiable personal data and therefore did not require formal ethical approval. The analysis was conducted on a publicly available, anonymized e‐commerce dataset. Nevertheless, the study recognizes broader ethical considerations relevant to data‐driven customer analytics, including responsible handling of customer‐related data, avoidance of privacy intrusion, and the need to monitor potential bias in predictive models when such frameworks are transferred to real deployment settings.

## Consent

The authors have nothing to report.

## Conflicts of Interest

The authors declare no conflicts of interest.

## Data Availability

The data that support the findings of this study are publicly available from the Brazilian E‐Commerce Dataset (Olist) via Kaggle. All codes used for analysis and model development are available from the corresponding author upon reasonable request.
